# Nutritional assessment among adult patients with suspected or confirmed active tuberculosis disease in rural India

**DOI:** 10.1371/journal.pone.0233306

**Published:** 2020-05-22

**Authors:** Elaine A. Yu, Julia L. Finkelstein, Patsy M. Brannon, Wesley Bonam, David G. Russell, Marshall J. Glesby, Saurabh Mehta

**Affiliations:** 1 Division of Nutritional Sciences, Cornell University, Ithaca, New York, United States of America; 2 Arogyavaram Medical Centre, Madanapalle, Andhra Pradesh, India; 3 Department of Microbiology and Immunology, College of Veterinary Medicine, Cornell University, Ithaca, New York, United States of America; 4 Weill Cornell Medical College, New York, New York, United States of America; 5 Institute for Nutritional Sciences, Global Health, and Technology, Cornell University, Ithaca, New York, United States of America; University of Botswana, BOTSWANA

## Abstract

**Objectives:**

Our study goal was to evaluate a set of nutritional indicators among adults with confirmed or suspected active tuberculosis disease in southern India, given the limited literature on this topic. Study objectives were to assess the: I) double burden of malnutrition at individual and population levels; II) relative performance of anthropometric indicators (body mass index, waist circumference) in diabetes screening; and III) associations between vitamin D and metabolic abnormalities.

**Design:**

Cross-sectional study.

**Setting:**

Hospital in rural southern India.

**Participants:**

Among adult patients (n = 834), we measured anthropometry, body composition, and biomarkers (vitamin D, glycated hemoglobin, hemoglobin) of nutritional status. Subsets of participants provided blood and sputum samples.

**Results:**

Among participants, 91.7% had ≥ 1 malnutrition indicator; 34.6% had both undernutrition and overnutrition indicators. Despite the fact that >80% of participants would be considered low-risk in diabetes screening based on low body mass index and waist circumference, approximately one-third had elevated glycated hemoglobin (≥ 5.7%). The lowest quintile of serum 25-hydroxyvitamin D was associated with an increased risk of glycated hemoglobin ≥ 5.7% (adjusted risk ratio 1.61 [95% CI 1.02, 2.56]) compared to the other quintiles, adjusting for age and trunk fat.

**Conclusions:**

Malnutrition and diabetes were prevalent in this patient population; since both can predict poor prognosis of active tuberculosis disease, including treatment outcomes and drug resistance, this emphasizes the importance of dual screening and management of under- and overnutrition-related indicators among patients with suspected or active tuberculosis disease. Further studies are needed to determine clinical implications of vitamin D as a potential modifiable risk factor in metabolic abnormalities, and whether population-specific body mass index and waist circumference cut-offs improve diabetes screening.

## Introduction

*Mycobacterium tuberculosis* (*M*. *tb*) causes the greatest number of deaths worldwide, compared to any other single infectious agent [1]. According to the World Health Organization (WHO), there were 1.7 billion people with latent tuberculosis (TB) infection, 10.0 million incident cases of active TB disease, and 1.2 million TB-related deaths in 2018 [1]. In India, there were nearly 2.7 million incident cases of active TB disease and 449,000 TB-related deaths in 2018 [1]. The burden of disease from active TB disease disproportionately affects low- and middle-income countries [2, 3]. Over 95% of reported TB cases were in 119 low- and middle-income countries [2].”

Active TB disease is associated with malnutrition risk factors, including undernutrition and overnutrition-related clinical sequelae (e.g. diabetes) [2–7]. Undernutrition and diabetes, respectively, are prevalent among patients with active TB disease, and increase the risk of progressing to active TB disease as well as worse TB treatment outcomes, including greater risk relapse and drug resistance [5–7]. Putative mechanisms at this complex nexus of undernutrition, diabetes, and active TB disease include that: inadequate nutrients adversely affect cell-mediated immunity, which is necessary in the human host response against *M*. *tb*; [8] active TB disease may alter metabolic processes, including increasing energy requirements and loss of appetite [7].

Nutritional screening, assessment, and management are recommended for all patients with TB during diagnosis, treatment, and management of active TB disease [7, 9]. However, there have been a limited number of comprehensive evaluations of nutritional status among patients with active TB disease in many resource-limited settings. Our study goal was to assess a panel of nutritional indicators among a patient population with confirmed or suspected active TB disease. Our three study objectives were based on prior literature. First, we assessed the prevalence of double burden of malnutrition (under- and over-nutrition indicators) at both the individual and population levels. Second, we evaluated the relative performance of anthropometric indicators (body mass index [BMI], waist circumference [WC]) in diabetes screening, compared to HbA1c. Last, we assessed the associations between vitamin D and metabolic abnormalities.

## Materials and methods

### Ethical conduct of research

The study protocol was designed in accordance with the Declaration of Helsinki principles, and approved by the Institutional Review Board at Cornell University and Institutional Ethics Committee at Arogyavaram Medical Centre. All study participants provided informed written consent to participate prior to data collection. Study participants had minimal risks or harms, largely associated with additional biological sample collection, aside from that associated with their routine clinical care.

### Study population

This cross-sectional study included a convenient consecutive sample of patients at a hospital (Arogyavaram Medical Centre) in Andhra Pradesh, India. The clinic is part of a hospital with inpatient and outpatient facilities, which regularly provides services for patients with suspected or confirmed active TB disease. Hospital physicians referred their patients with suspected active TB disease to study staff. Study participants (n = 834) were recruited and sequentially enrolled during their hospital visits between September 2014 and May 2016. The total sample size was based on all participants who were enrolled during the window of data collection dates.

### Data collection

Trained research assistants administered structured interviews to collect sociodemographic and clinical data in the local language, Telugu. Sociodemographic covariates included: age, sex, educational level, monthly household income, and cigarette use. A study physician conducted a complete examination, including blood pressure measurements. Data were collected based on the study protocol, including data management plans (e.g. quality control via skip patterns in electronic data collection forms, data confidentiality).

Subsets of study participants provided sputum and blood samples, based on their hospital visit and the recommendations of their physicians. Trained phlebotomists collected blood samples using standard clinical protocols. From this convenient sample, available blood samples were assayed for serum 25-hydroxyvitamin D (25[OH]D; n = 156) and glycated hemoglobin (HbA1c; n = 236). For active TB disease assessment (n = 363), patients provided a sputum sample at the time of his or her initial hospital visit, and a second sputum sample on the following morning.

### Laboratory analyses

Each sputum sample was assessed for active TB disease by the detection of standard acid-fast bacilli (AFB) with Ziehl-Neelsen staining and conventional light microscopy. Blood samples were assayed for HbA1c (%) by high-performance liquid chromatography (D-10; Bio-Rad Laboratories, Hercules, California, United States [US]). Serum 25-hydroxyvitamin D (25[OH]D; nmol/L) was assayed by chemiluminescence immunoassay (LIAISON; DiaSorin Inc., Stillwater, Minnesota, US). We also participated in the D External Quality Assurance Scheme (www.deqas.org) program; compared to the National Institute of Standards and Technologies target values, our median percentage difference was -8.8 (IQR -19.7, -4.4). Complete blood counts were assessed by an automated hematology analyzer (BC-2800; Mindray Bio-Medical Electronics Co., Ltd., Shenzhen, People’s Republic of China).

### Anthropometry

Anthropometric measurements were recorded, based on World Health Organization (WHO) recommendations and other commonly utilized standard methods [10, 11]. Height, WC, mid-upper arm circumference (MUAC), and skinfold thickness were measured to the nearest 0.1 centimeter; weight was assessed to the nearest 0.1 kilogram. Total body and trunk fat (%) were assessed by bioelectrical impedance analysis (BC-418 MA; Tanita Corporation, Tokyo, Japan; 8 electrode).

### Definitions

Anthropometric measurements (BMI, WC) were considered as continuous and categorized variables. BMI was categorized per standard WHO cut-offs (underweight < 18.5 kg/m^2^; normal weight ≥ 18.5 and < 25.0 kg/m^2^; overweight ≥ 25.0 and < 30.0 kg/m^2^; and obese ≥ 30.0 kg/m^2^) [12]. Additionally, we considered BMI cut-offs for Asian populations (≥ 18.5 to < 23.0 kg/m^2^; ≥ 23.0 to < 27.5 kg/m^2^; ≥ 27.5 kg/m^2^), based on a WHO expert consultation [13]. WC was considered as a continuous variable and tertiles based on distribution in this population. Elevated WC was also defined based on the International Diabetes Federation (IDF) cut-offs (men ≥90 cm, women ≥80 cm) recommended for individuals in South Asia [14]. We calculated limb fat as the sum of body fat (kg) in all four limbs.

Hemoglobin (g/L) was considered as a continuous and categorical variables (including quintiles). Biologically implausible values were considered missing. Anemia and severe anemia were defined by hemoglobin cut-offs, per WHO recommendations (S1 Table) [15]. Hemoglobin was adjusted for smoking by subtracting 0.3 g/L among any individuals who self-reported as currently smoking [15]. We also considered other factors that affect anemia (e.g. pregnancy, residential elevation above sea level, smoking) [15]. However, no study participants self-reported pregnancy; and all study participants resided near the hospital study site, which had an elevation that did not require altitude adjustment [15].

Red blood cell morphology were categorized to assess abnormalities, which reflect different causes of anemia and commonly assist in evaluating different types of anemia [16]. Microcytosis (<80 femtoliters/cell) was defined as mean corpuscular volume (MCV; femtoliters/cell; S1 Table) [16]. Mean corpuscular hemoglobin (MCH; picograms/cell) was categorized as hyper-, normo-, and hypochromia (S1 Table) [16]. Hypochromic microcytic anemia was defined by hemoglobin (men <130 g/L, women <120 g/L) [15] as well as MCV <80 femtoliters/cell and MCH <27 picograms/cell (S1 Table) [16]. Elevated erythrocyte sedimentation rate (ESR; mm/hr) was categorized according to age- and sex-specific cut-off values (S1 Table) [17, 18]. Biologically implausible values were considered missing; additionally, statistical outliers (<2.5^th^ and >97.5^th^ percentiles) were excluded.

HbA1c was categorized by common cut-off values (≥ 6.5%, ≥ 5.7% to < 6.5%, and <5.7%) that are recommended as part of the clinical diagnostic criteria of diabetes and pre-diabetes [19, 20]. Active TB disease was defined as at least one positive AFB sputum smear result. This included patients with one positive AFB result (regardless of first or second sputum sample), as well as two positive AFB results, according to the standard active TB disease diagnostic guidelines in India [21]. Vitamin D status was defined with several cut-off values (25[OH]D <25.0 nmol/L [22], <40.0 nmol/L [23], <50.0 nmol/L [23, 24], <75.0 nmol/L [24]). Additionally, we categorized 25(OH)D (nmol/L) by quintiles.

Educational level was categorized based on self-reported completion of formal coursework (i.e., no formal education or illiterate; primary [grades 1–5], secondary [grades 6–12, including higher secondary], and any higher education [college, graduate, post-graduate]). Self-reported monthly household income was dichotomized as < 5000 Indian rupees (INR) or ≥ 5000 INR) [25]. This cut-off was rounded from the cut-off of 4860 INR, based on the estimated monthly consumption expenditure for a family of five residing in a rural area (2011–2012 prices) and the national poverty line of India (Government of India, Planning Commission, 2014 Report) [25]. Cigarette use was categorized as current, previous, or never.

### Statistical analyses

Continuous variables were assessed for normality using the Shapiro-Wilk test statistic. For descriptive statistics, continuous variables were reported as medians (interquartile ranges [IQRs]); categorical variables were reported as percentages. Subgroup comparisons were based on tests for continuous (i.e., Kruskal-Wallis) and categorical variables (i.e., likelihood ratio test).

We assessed associations between anthropometric indicators and HbA1c with univariate and multivariate linear and binomial regressions. Associations between anthropometric screening indicators (BMI, WC) and HbA1c ≥ 6.5% were assessed by multivariate log-binomial regressions; key covariates (age, sex) were accounted for in these models. The predictive performance of anthropometric screening indicators (i.e., BMI, WC; categorical variables) for elevated HbA1c (≥ 6.5%) were assessed by sensitivity, specificity, positive predictive value (PPV), negative predictive value (NPV), and receiver operating characteristic (ROC) curve analysis [26]. Observed area under the ROC curves (AUCs) were compared to AUC for the null hypothesis (0.5), based on the contrast matrices of differences between the areas under the ROC curves (ROCCONTRAST in SAS statistical software) [27]. We also examined the predictive performance of anthropometric screening indicators, considering a cut-off of HbA1c ≥ 5.7% as the outcome of interest.

For the associations between vitamin D and metabolic abnormality outcomes, the selection of potential confounders was based on approaches suggested by Rothman and Greenland [28]. In brief, we identified known or suspected risk factors based on *a priori* literature review [29, 30]. For each association of interest, we included the confounders in the final adjusted model based on a 10% change-in-estimate criterion [28]. For binary outcomes, binomial regressions were utilized when models converged. Modified Poisson estimates were utilized if binomial models failed to converge [31]. The analytical subsets were as follows (for each respective outcome): WC (n = 150), HbA1c (n = 149), and blood pressure (n = 99); missing-data indicators were utilized for covariates with missingness [28].

Statistical analyses were conducted with SAS statistical software (version 9.4; SAS Institute Incorporated, Cary, North Carolina). All comparisons were two-sided; and considered statistically significant with an α value of 0.05.

### Patient involvement

Prior to data collection, patients provided input regarding the structured interview questions; some of their feedback was integrated in the final data collection forms. These patients were individuals who visited and resided near a hospital in rural southern India, and the source population of our study.

## Results

### Sociodemographic and clinical characteristics

The median age of study participants was 48 years (IQR 35, 60; Table 1), including individuals ranging from 18 to 87 years of age. Nearly three quarters of study participants were male (n = 610; Table 1). Over half of study participants (54.0%) had a self-reported monthly household income less than 5000 INR (Table 1). Among study participants with AFB assessment (n = 363), 24.2% had active TB disease (Table 1).

**Table 1:** Sociodemographic and clinical characteristics of study participants ^a^.

	Total n = 834	Men n = 610 (73.1%)	Women n = 224 (26.9%)	p ^b^
***Sociodemographic characteristics***				
Age (years), median (IQR)	48 (35, 60)	51 (37, 64)	40 (30, 53)	<0.01 ^c^
Education (completed level/class), n (%)				
Illiterate	404 (48.4%)	272 (44.6%)	132 (58.9%)	0.03 ^d^
Primary ^g^	209 (25.1%)	183 (30.0%)	26 (11.6%)	
Secondary, middle	152 (18.2%)	103 (16.9%)	49 (21.9%)	
Graduate, diploma, post-graduate	69 (8.3%)	52 (8.5%)	17 (7.6%)	
Monthly household income < 5000 INR, n (%) ^h^	354 (54.0%)	231 (50.7%)	123 (61.5%)	0.01 ^d^
Self-reported current cigarette smoking, n (%)	41 (4.9%)	41 (6.7%)	0 (0.0%)	— ^f^
***Clinical characteristics***				
Active TB disease, n (%) ^i^	88 (24.2%)	80 (27.7%)	8 (10.8%)	<0.01 ^d^
Coughing, n (%) ^j^	693 (85.9%)	516 (87.9%)	177 (80.5%)	0.01 ^d^
With sputum production ^j^^,^ ^k^	511 (74.2%)	394 (77.0%)	117 (66.1%)	0.01 ^d^
With blood ^j^^,^ ^k^	72 (10.5%)	59 (11.6%)	13 (7.4%)	0.11 ^d^
Fever, n (%)	331 (41.2%)	246 (42.1%)	85 (39.0%)	0.43 ^d^
Night sweats, n (%)	127 (15.7%)	91 (15.5%)	36 (16.4%)	0.76 ^d^

AFB, acid-fast bacilli; IDF, International Diabetes Federation; INR, Indian rupees; TB, tuberculosis; WHO, World Health Organization

^a^ Among study participants with available hemoglobin, age, and sex data (n = 834). Covariates with missing observations included: monthly household income (n = 178), cigarette smoking (n = 2), active TB disease assessment (n = 471), coughing (n = 27), sputum production with cough (n = 145), blood with cough (n = 151), fever (n = 31), night sweats (n = 26).

^b^ Comparison of each sociodemographic or clinical indicator between men and women

^c^ Hodges Lehmann estimator (two-sided normal approximation based on Wilcoxon sign rank test).

^d^ Likelihood ratio test

^e^ Kruskal Wallis test. Normality assumptions not met based on Shapiro-Wilk test statistic.

^f^ Sample cell size < 5

^g^ Including completed and some primary education

^h^ Cut-off value of 5000 INR rounded from the cut-off of 4860 INR, which is based on monthly consumption expenditure for a family of five residing in a rural area (2011–12 prices), per the India national poverty line (Government of India, Planning Commission, 2014 Report) [25]

^i^ Based on AFB sputum smear microscopy.

^j^ According to self-report

^k^ Among those with cough

Overall, median systolic blood pressure was 110 mmHg (IQR 100, 120); 11.1% of study participants had systolic blood pressure ≥ 140 mmHg. Median diastolic blood pressure was 70 mmHg (IQR 60, 80); 13.1% of patients had elevated diastolic blood pressure (≥ 90 mmHg). Eight percent of patients had abnormal blood pressure (either elevated systolic, diastolic, or both).

### Anthropometry

Per the WHO categories for BMI, 46.8% of men and 40.2% of women were considered underweight (BMI < 18.5 kg/m^2^; Table 2). Only 9.0% of men and 19.6% of women were considered overweight or obese (BMI ≥ 25.0; Table 2). Based on the alternative WHO BMI cut-offs recommended for Asian populations, [13] 83.1% of men and 69.2% of women were underweight or normal weight (BMI < 23.0 kg/m^2^; Table 2). Median WC was 70.3 cm (IQR 65.0, 80.1) among men and 66.9 cm (IQR 60.7, 77.5) among women (p<0.01; Table 2). Overall, 87.5% of study participants were below the IDF WC cut-off (men 90.7%, women 78.5%; p<0.01; Table 2). Additional anthropometry and body composition measurements are in S2 Table.

**Table 2:** Anthropometry among adults with suspected or confirmed active TB disease ^a^.

		Total n = 834	Men n = 610 (73.1%)	Women n = 224 (26.9%)	p ^b^
BMI (kg/m^2^), median (IQR)		18.8 (16.8, 22.2)	18.6 (16.8, 21.5)	19.6 (16.9, 23.8)	<0.01 ^d^
*WHO standard categories*, *n (%)*	<18.5	357 (45.0%)	271 (46.8%)	86 (40.2%)	< 0.01 ^c^
	≥ 18.5 to < 25.0	342 (43.1%)	256 (44.2%)	86 (40.2%)	
	≥ 25.0 to < 30.0	72 (9.1%)	45 (7.8%)	27 (12.6%)	
	≥ 30.0	22 (2.8%)	7 (1.2%)	15 (7.0%)	
*WHO alternative categories*, *n (%)*	<18.5	357 (45.0%)	271 (46.8%)	86 (40.2%)	< 0.01 ^c^
	≥ 18.5 to < 23.0	272 (34.3%)	210 (36.3%)	62 (29.0%)	
	≥ 23.0 to < 27.5	121 (15.3%)	82 (14.2%)	39 (18.2%)	
	≥ 27.5	43 (5.4%)	16 (2.8%)	27 (12.6%)	
WC (cm), median (IQR)		69.5 (63.4, 79.6)	70.3 (65.0, 80.1)	66.9 (60.7, 77.5)	<0.01 ^d^
< IDF cut-off, n (%) ^e^		700 (87.5%)	536 (90.7%)	164 (78.5%)	< 0.01 ^c^

BMI, body mass index; IDF, International Diabetes Federation; WC, waist circumference; WHO, World Health Organization

^a^ Among study participants with available hemoglobin, age, and sex data (n = 834). Covariates with missing observations included: BMI (n = 41), WC (n = 34).

^b^ Comparison of each anthropometric indicator between men and women

^c^ Likelihood ratio test

^d^ Kruskal Wallis test. Normality assumptions not met based on Shapiro-Wilk test statistic.

^e^ IDF WC cut-off values among South Asian populations (men <80 cm, women <90 cm)

### Anemia

Median hemoglobin concentrations were 115.0 g/L (IQR 99.0, 129.7; Table 3). Among study participants, 71.9% (n = 600) had anemia and 6.4% had severe anemia (n = 53; Table 3). The prevalence of anemia was 82.1% in women (n = 184), and 68.2% in men (n = 416; Table 3). Elevated ESR was observed in 59.0% of participants (n = 492; Table 3).

**Table 3:** Biochemical indicators of study participants ^a^.

	Total n = 834	Men n = 610 (73.1%)	Women n = 224 (26.9%)	p ^b^
***Median (IQR) or n (%)***				
Hemoglobin (g/L) ^c^	115.0 (99.0, 129.7)	120.0 (103.0, 132.7)	102.0 (90.0, 115.0)	<0.01 ^i^
Anemia ^d^	600 (71.9%)	416 (68.2%)	184 (82.1%)	<0.01 ^j^
Severe anemia ^d^	53 (6.4%)	32 (5.3%)	21 (9.4%)	0.04 ^j^
Hypochromic microcytic anemia ^d^^,^ ^e^	175 (23.0%)	108 (19.2%)	67 (34.2%)	<0.01 ^j^
HbA1c (%)	5.4 (5.0, 5.8)	5.4 (5.1, 5.8)	5.3 (4.9, 5.7)	0.07 ^i^
≥ 6.5%	29 (12.3%)	21 (13.0%)	8 (10.7%)	0.20 ^j^
< 6.5% and ≥ 5.7%	48 (20.3%)	36 (22.4%)	12 (16.0%)	
< 5.7%	159 (67.4%)	104 (64.6%)	55 (73.3%)	
25(OH)D (nmol/L)	51.8 (36.0, 70.0)	55.2 (38.8, 75.0)	48.4 (31.7, 64.2)	0.04 ^i^
<25 ^f^	17 (10.9%)	10 (10.0%)	7 (12.5%)	0.64 ^j^
< 40 ^g^	49 (31.4%)	26 (26.0%)	23 (41.1%)	0.05 ^j^
<50 ^g^^,^^h^	73 (46.8%)	43 (43.0%)	30 (53.6%)	0.20 ^j^
<75 ^h^	126 (80.8%)	75 (75.0%)	51 (91.1%)	0.01 ^j^
ESR (mm/hr)	30 (15, 55)	30 (12, 60)	25 (15, 45)	0.31 ^i^
Elevated ESR ^d^	492 (59.0%)	375 (61.5%)	117 (52.2%)	0.02 ^j^
White blood cell count (cells/cmm)	9,500 (7,740, 12,300)	9,700 (7,900, 12,380)	9,200 (7,300, 12,100)	0.03 ^i^
Elevated WBC (>11,000)	274 (34.4%)	213 (36.4%)	61 (28.9%)	0.05 ^j^
Differential count (%)				
Neutrophils	68 (60, 76)	70.0 (61.0, 77.0)	64.0 (57.0, 72.5)	<0.01 ^j^
Lymphocytes	23 (16, 31)	22 (15, 30)	27.5 (20.0, 34.0)	<0.01 ^j^
Monocytes	4 (3, 5)	4 (3, 5)	4 (3, 5)	0.73 ^j^
Eosinophils	4 (3, 5)	4 (3, 5)	4 (3, 5)	0.56 ^j^

25(OH)D, 25-hydroxyvitamin D; IQR, interquartile range; HbA1c, glycated hemoglobin; ESR, erythrocyte sedimentation rate; WBC, white blood cells

^a^ Among study participants with available anemia (hemoglobin) data (n = 834); of this total, covariates with missing observations included: hypochromic microcytic anemia (74 missing observations), white blood cell count (38 missing observations), lymphocytes (1 missing observation). Two sub-analyses were among sample subsets with 25(OH)D (n = 156) and HbA1c (n = 236) data.

^b^ Comparison of each biochemical indicator by biological sex

^c^ Hemoglobin adjusted for smoking, based on WHO recommendations. 0.3 g/L hemoglobin was subtracted among any individuals who self-reported as currently smoking [15].

^d^ See definitions in S1 Table

^e^ Among those with anemia and available data for hypochromia and microcytosis

^f^ Scientific Advisory Council of Nutrition cut-off value recommended to prevent rickets

^g^ Institute of Medicine cut-off values for 25(OH)D deficiency and insufficiency among healthy populations

^h^ Endocrine Society cut-off values for 25(OH)D deficiency and insufficiency among populations at risk of vitamin D deficiency

^i^ Hodges Lehmann estimator (two-sided normal approximation based on Wilcoxon sign rank test). Normality assumptions not met based on Shapiro-Wilk test statistic.

^j^ Likelihood ratio test

### Glycemic status

Overall, 12.3% of study participants had HbA1c ≥ 6.5%; and 20.3% of participants had HbA1c ≥ 5.7% and < 6.5% (Table 3). Median HbA1c was 5.4% (IQR 5.0, 5.8; Table 3), and similar between men and women (p>0.05; Table 3).

### Double burden of malnutrition

Nearly all (91.7%) study participants had at least one malnutrition indicator, based on BMI, anemia, MUAC, WC (Table 4). Among participants, 66.4% had at least two malnutrition indicators; 27.6% had three or more malnutrition indicators. Among study participants with both undernutrition and overnutrition indicators (34.6%; Table 4), 82.7% had HbA1c ≥ 5.7%, 96.0% had anemia, 45.3% were underweight, and 14.7% were overweight or obese.

**Table 4:** Double burden of malnutrition (n = 217).

		**Undernutrition Indicators** ^a^^,^ ^c^	
		Y	N
**Overnutrition Indicators** ^b^^,^ ^c^	Y	75 (34.6%)	16 (7.4%)
	N	108 (49.8%)	18 (8.3%)

BMI, body mass index; WC, waist circumference; IDF, International Diabetes Federation; MUAC, mid-upper arm circumference

^a^ Includes any assessed indicator of undernutrition (BMI <18.5 kg/m^2^, anemia, or low MUAC)

^b^ Includes any assessed indicator of overnutrition (BMI ≥ 25.0 kg/m^2^, or WC ≥ IDF cutoff) or diet-related non-communicable disease (HbA1c ≥ 5.7%)

^c^ Based on available data for all variables considered (BMI, WC, MUAC, hemoglobin, HbA1c). A complete case analysis approach was utilized. If any of these variables were missing, the observation was considered missing.

### Diabetes screening performance of anthropometric indicators

Sensitivity, specificity, PPV and NPV of anthropometric indicators (BMI [standard and alternative WHO categorizations], and WC [IDF cut-offs]) of HbA1c ≥ 6.5% are in Table 5. Considering BMI (standard WHO categories) as an indicator for HbA1c ≥ 6.5%, the AUC was 0.58 among all study participants (p = 0.25), sensitivity was 0.21 (95% CI: 0.06, 0.35), and specificity was 0.92 (95% CI: 0.88, 0.96; Table 5).

**Table 5:** Comparison of anthropometric (BMI, WC) screening cut-offs for HbA1c ≥ 6.5% ^a^^,^ ^b^.

	HbA1c						
BMI ^c^ (kg/m^2^)	≥6.5%	<6.5%		Sensitivity (95% CI)	Specificity (95% CI)	PPV (95% CI)	NPV (95% CI)
≥ 25.0	6	16	22				
< 25.0	23	178	201	0.21 (0.06, 0.35)	0.92 (0.88, 0.96)	0.27 (0.09, 0.46)	0.89 (0.84, 0.93)
	29	194	223				
≥ 23.0	9	29	38				
< 23.0	20	165	185	0.31 (0.14, 0.48)	0.85 (0.80, 0.90)	0.24 (0.10, 0.37)	0.890.85, 0.94)
	29	194	223				
WC ^d^ (cm)							
≥ IDF cut-off	8	24	32				
< IDF cut-off	20	177	197	0.29 (0.12, 0.45)	0.88 (0.84, 0.93)	0.25 (0.10, 0.40)	0.90 (0.86, 0.94)
	28	201	229				

BMI, body mass index; WC, waist circumference; HbA1c, glycated hemoglobin; PPV, positive predictive value; NPV, negative predictive value; CI, confidence interval; IDF, International Diabetes Federation; WHO, World Health Organization

^a^ Among study participants with available data (hemoglobin, HbA1c, as well as either BMI [categorical; n = 223] or WC [n = 229]).

^b^ American Diabetes Association cut-points of HbA1c ≥ 6.5%.

^c^ WHO classifications (standard and alternative categorization for Asian populations)

^d^ IDF WC cut-off values among South Asian populations (men ≥80 cm, women ≥90 cm)

We also considered the predictive performance of anthropometric indicators for elevated HbA1c, using a cut-off of ≥ 5.7% as the outcome of interest (S3 Table). With BMI (standard WHO categories) as the indicator, the AUCs were: overall 0.49 [p = 0.76], men 0.54 [p = 0.58], women 0.57 [p = 0.17]). The AUC for WC were 0.57 (p = 0.07) among all study participants, 0.53 (p = 0.36) among men, and 0.61 (p = 0.09) among women.

WC (adjusted risk ratio [aRR] 1.03 [95% CI 1.01, 1.06]; continuous variable) was associated with HbA1c ≥ 6.5%, adjusting for age and sex. Similarly, individuals in the highest tertile of WC were positively associated with HbA1c ≥ 6.5% (Tertile 3 vs 1: aRR 2.87 [95% CI 1.19, 6.92]), compared to those in the lowest WC tertile, controlling for age and sex. Higher BMI (≥ 25.0 kg/m^2^) was positively associated with HbA1c (≥ 6.5%; p<0.01), accounting for age and sex.

### Associations between vitamin D and metabolic abnormalities

Median serum 25(OH)D was 51.8 nmol/L (IQR 36.0, 70.0; Table 3). Across quintiles, the median 25(OH)D concentration was 24.9 nmol/L (IQR 19.1, 28.7), 38.8 nmol/L (IQR 35.4, 42.3), 51.7 nmol/L (IQR 49.0, 55.2), 67.5 nmol/L (IQR 63.1, 70.0), and 82.9 nmol/L (IQR 76.9, 102.1).

25(OH)D < 50 nmol/L was associated with HbA1c (%; p = 0.04), adjusting for age and fat free mass (Table 6). The lowest quintile of serum 25(OH)D was associated with an increased risk of HbA1c ≥ 5.7% (aRR 1.61 [95% CI 1.02, 2.56]) compared to the other quintiles, adjusting for age and trunk fat (Table 6). However, the other associations that were assessed between 25(OH)D (continuous, <50 nmol/L) were not associated with HbA1c (continuous, ≥ 6.5% or ≥ 5.7%; p>0.05; Table 6).

**Table 6:** Serum 25-hydroxyvitamin D and glycated hemoglobin (n = 149).

Vitamin D (25[OH]D)	HbA1c (continuous; linear regression)				HbA1c (categorical ≥ 6.5%; binomial regression) ^a^^,^ ^c^				HbA1c (categorical ≥ 5.7%; binomial regression) ^a^^,^ ^c^			
	Unadjusted		Adjusted ^b^		Unadjusted		Adjusted ^b^		Unadjusted		Adjusted ^b^	
	β (SE)	p	β (SE)	p	RR	95% CI	RR	95% CI	RR	95% CI	RR	95% CI
Continuous (nmol/L)	<-0.01 (<0.01)	0.48	<-0.01 (<0.01)	0.27	1.00	0.98, 1.01	0.99 ^d^	0.97, 1.01	0.99	0.98, 1.01	0.99	0.98, 1.00
< 50 nmol/L (Endocrine Society)	0.40 (0.27)	0.13	0.54 (0.27)	0.04	1.57	0.63, 3.90	1.80 ^d^	0.67, 4.84	1.36	0.84, 2.19	1.54	0.98, 2.41
Quintiles (low 1 vs 2–5)	0.09 (0.33)	0.79	0.21 (0.33)	0.52	0.82	0.25, 2.66	0.92 ^d^	0.26, 3.34	1.46	0.88, 2.40	1.61	1.02, 2.56

^a^ HbA1c cut-offs based on WHO and IDF recommendations

^b^ We considered known or suspected risk factors for HbA1c as potential confounders. These potential confounders were included if p<0.25 from univariate regressions (linear or binomial regression model beta coefficients; likelihood ratio tests). Based on a change in estimate approach, covariates were included in the final adjusted model if they changed the estimate by ≥10%. The final covariates for the association of HbA1c (categorical, ≥5.7%) and vitamin D (quintiles 1 vs 2–5) were utilized in final models in this table; these included: age, trunk fat. Analysis among participants with available HbA1c and vitamin D data (n = 149). Missing-data indicators were used for covariates with missingness.

^c^ Binomial regression unless otherwise stated

^d^ Poisson regression due to no model convergence

25(OH)D was not associated with elevated WC (above the IDF cut-off values) in multivariate linear and binomial (or Poisson) regression models (p>0.05; S4 Table). The considered associations between 25(OH)D (continuous, <50 nmol/L) were not associated with blood pressure, including systolic (continuous, elevated), diastolic (continuous, elevated), and abnormally high blood pressure (p>0.05; S5 Table).

## Discussion

Our results showed a high prevalence of malnutrition among a patient population with recently confirmed or suspected active TB disease. Over 90% of participants had at least one malnutrition indicator; one in three participants had both undernutrition and overnutrition indicators. Despite the fact that more than 80% of participants would be considered low-risk in diabetes screening, based on low BMI and WC, approximately one-third had elevated HbA1c (≥ 5.7%). Common cut-off values for anthropometry (overnutrition indicators) had suboptimal predictive performance in detecting elevated HbA1c among an adult outpatient population with a high prevalence of low BMI. Findings suggest the need for population-specific cut-offs for BMI and WC, given that each remained respectively associated with HbA1c. Lastly, vitamin D status and HbA1c were inversely associated.

### Undernutrition and elevated HbA1c among patients with suspected or confirmed active tuberculosis disease

The prevalence of underweight (45.0%; BMI <18.5 kg/m^2^) in our study population was substantially higher than prior South Asia (<25.0%) and global (<12.5%) estimates for adults [32], though other estimates among patients with active TB disease in India have ranged widely. In a study among individuals with active TB disease in Chhattisgarh, over 80% of men and 90% of women had BMI < 18.5 kg/m^2^ prior to anti-TB treatment [33], which was higher than among our participants. Another study in Karnataka found that 30% of patients with pulmonary or extrapulmonary active TB disease had BMI <18.4 kg/m^2^ [34]. Our finding that 2.8% of study participants had BMI ≥ 30 kg/m^2^ was lower than WHO obesity estimates for India (4.9%) and globally (11% men, 15% women) [35].

Based on the WHO classification, the public health significance of anemia in our study population (82.1% women; 68.2% men) is considered severe [15]. A previous global estimate of anemia prevalence was 24.8% [36], which is less than half the proportion observed among our study participants with suspected or confirmed active TB disease. Moreover, in contrast to the global estimate of anemia among women of reproductive age (29.4% (95% CI: 24.5, 35.0) [37], four of every five females in our study had anemia. Previous literature has corroborated the anemia of inflammation, including in active TB disease [38], however nationally representative estimates of anemia prevalence among people with active TB disease are limited, particularly in low- and middle-income countries.

Globally, nearly one of every ten (8.5%) adults has diabetes [39]. In the WHO South-East Asia region, diabetes prevalence among adults is 8.6% [39]. Among our study participants, 12.3% had elevated HbA1c ≥6.5%, despite the high prevalence of people with BMI <25 kg/m^2^. Our results confirmed a high prevalence of malnutrition among patients with confirmed or suspected active TB disease; over nine of every ten study participants had at least one indicator of malnutrition. Moreover, approximately one-third of study participants had indicators of both undernutrition and overnutrition, suggesting these patients are affected by the double burden of malnutrition at the individual-level.

### Relative performance of anthropometric indicators in diabetes screening

Higher BMI (overweight and obesity) and WC are well-established modifiable risk factors of type 2 diabetes mellitus (T2DM) [14, 20, 39–42]. The respective associations between BMI and WC with elevated HbA1c have been confirmed in several studies among populations in North America [41, 43] and Asia (India [44, 45], China [46]). One US study among Mexican Americans found an 11 times increased risk of non-insulin dependent diabetes mellitus among those with WC in the highest quartile, compared to the lowest quartile [43]. Despite the high prevalence of low BMI in our study population, BMI and WC similarly were associated with elevated HbA1c.

International and national public health entities, including the WHO [39] and IDF [14], recommend common cut-off values of elevated BMI and WC that identify individuals at risk for T2DM. However, the observation of higher diabetes prevalence among populations with lower mean BMI has instigated the question of whether population-specific cut-off values of anthropometric indicators would be more appropriate for diabetes screening in some populations [13, 47–50], such as India.

Previous studies have shown a wide heterogeneity of predictive performance of anthropometric indicators in diabetes and pre-diabetes screening. For example, in one US study among 12,814 adults (African American, white), areas under the ROC curves were similar for BMI (African American men 0.69, white men 0.70; African American women 0.66, white women 0.72) and WC (African American men 0.70, white men 0.70; African American women 0.69, White women 0.73) in predicting diabetes [51].

A growing body of evidence suggests research gaps and limitations in the predictive performance of commonly used anthropometric indicator cut-offs for T2DM screening. First, the heterogeneity of body fat distribution is hypothesized to affect T2DM risk [52], which could cause common cut-points of anthropometric screening indicators for diabetes to perform worse (more false negatives or positives) in some populations. Central obesity as well as visceral fat have been more strongly associated with insulin resistance and T2DM, relative to overall obesity [53–55]. Studies have demonstrated that individuals with similar BMI sometimes differ substantially in body fat distribution and percentage [50, 56, 57], metabolic syndrome [58], T2DM [59, 60]. As an example, the predisposition for central fat accumulation among Asian populations has been observed to differ from Caucasians [61], which could explain differential T2DM risk among individuals with the same BMI [13].

Second, studies have begun elucidating the biological basis for these observed patterns. At the cellular level, functional metabolic differences between adipocytes (brown, white, and beige [brown in white]) have been characterized [62–64]. Critically, studies have shown metabolically active brown adipocytes associated with improved T2DM indicators and lower BMI [63, 65]; in contrast, white adipocytes were associated with visceral fat, which has been linked to insulin resistance [66].

Successfully addressing the diabetes epidemic requires considering effective screening among populations with different body composition patterns. Overall in our study, common cut-off values for anthropometric screening indicators had suboptimal predictive performance in detecting elevated HbA1c among an adult outpatient population with lower adiposity. Findings suggest the need for population-specific cut-offs for BMI and WC, given that: 1) each remained respectively associated with elevated HbA1c; and 2) standard cut-offs misclassify the HbA1c status of many study participants.

Although our results reveal several research gaps, the importance and challenges of determining appropriate population-specific cut-off values of anthropometric indicators have been acknowledged in previous literature [47–50] and by a WHO expert consultation [13]. Future research questions include: What are the appropriate cut-offs across racial and ethnic subgroups, based on representative samples with external validity? How do different fat distributions (including differing body fat percentage and adipocyte type) affect the risk of T2DM incidence and severity? What are the cellular mechanisms involving different adipocyte types that contribute to T2DM development and progression?

### Vitamin D as a modifiable risk factor of metabolic indicators

Previous literature has found an inverse association between vitamin D status (25[OH]D concentration) and HbA1c [67, 68], which was consistent with our result. In a nationally representative study among adults in the US, the prevalence of high HbA1c (>6.0%) linearly decreased across vitamin D quintiles (p<0.01) [68]. Separately, systematic reviews have shown vitamin D supplementation was associated with HbA1c in some studies [69, 70]. Although other studies observed null results [71–73], many differed widely in methodology, including vitamin D dosage (duration, frequency, dosage).

Our finding that serum 25(OH)D was inversely associated with WC, which has been corroborated by other studies [74–76]. At the cellular level, other key findings that support epidemiological findings include the: a) isolation of vitamin D receptor (VDR) as well as hydroxylating enzymes of vitamin D in adipose tissues; b) storage and release of vitamin D in adipocytes [77–82]. Many questions remain, in order to elucidate the etiology and mechanisms of 25(OH)D in the context of adiposity and energy homeostasis [83], including the: extent of differences in vitamin D metabolism (*e*.*g*. VDR signaling, vitamin D activation:inactivation ratio, interactions with lipid-mediated regulatory processes such as via peroxisome proliferator-activated receptor gamma) across adipocyte types and heterogeneous body composition.

While our study demonstrated a null association between low 25(OH)D concentration and high systolic blood pressure, prior literature has supported an inverse association between 25(OH)D and the renin-angiotensin-aldosterone-system (RAAS), which regulates hypertension [84–86]. One hypothesized mechanism is that elevated vitamin D inhibits renin and angiotensin expression, which dampens the RAAS activity and subsequently decreases blood pressure [85–88]. Additionally, an overview of systematic reviews of vitamin D supplementation randomized controlled trials found that among nine meta-analyses, two showed protective effects of vitamin D supplements on blood pressure and six had null findings [89].

### Strengths and limitations

In our study, there were several strengths, including the: sample size, assessment of multiple BMI and WC categories (based on widely used cut-off values and population distribution [quantiles]), evaluation of microcytosis and hypochromia.

This study had several limitations, including the: cross-sectional study design (with a single timepoint assessment); potential residual confounding; external validity (generalizability of findings, especially among healthy populations); assessment of additional causes of low hemoglobin; biological samples obtained per standard of care (only from participants with a clinical indication and not collected at random or from all participants); and limited biomarker data (such as diagnoses of human immunodeficiency virus [HIV] and no peripheral smears to determine iron deficiency anemia). Active TB disease and diabetes have bi-directional impacts that we were not able to assess, particularly as we only measured HbA1c and not diabetes mellitus [90]. Iron deficiency with and without anemia has been associated with increased HbA1c [91], and this potential interaction needs be evaluated in this study population.

## Conclusions

In summary, our findings confirmed that malnutrition and elevated HbA1c were prevalent among this patient population with suspected and confirmed active TB disease in rural India. Dual screening and management of under- and overnutrition-related indicators are needed among patient populations with confirmed and suspected active TB disease, in order to facilitate improved TB control efforts. Further studies are needed to determine any clinical implications of the potential role of vitamin D as a modifiable risk factor in metabolic abnormalities, as well as whether population-specific BMI and WC cut-offs are needed among specific populations (e.g. metabolically unhealthy normal or underweight).

## Supporting information

S1 Table Definitions of anemia and related red blood cell indices. (DOCX)

S2 Table Additional anthropometric indicators among men and women. (DOCX)

S3 Table Comparison of anthropometric (BMI, WC) screening cut-offs for HbA1c ≥ 5.7%. (DOCX)

S4 Table Serum 25-hydroxyvitamin D and waist circumference (n = 150). (DOCX)

S5 Table Serum 25-hydroxyvitamin D and blood pressure (n = 99). (DOCX)

## Abbreviations

25(OH)D: 25-hydroxyvitamin D
AFB: acid-fast bacilli
aRR: adjusted risk ratio
AUC: area under the receiver operating characteristic curve
BMI: body mass index
ESR: erythrocyte sedimentation rate
HbA1c: glycated hemoglobin
HIV: human immunodeficiency virus
IDF: International Diabetes Federation
INR: Indian rupees
IQR: interquartile range
*M*. *tb*: *Mycobacterium tuberculosis*
MCH: mean corpuscular hemoglobin
MCV: mean corpuscular volume
MUAC: mid-upper arm circumference
NPV: negative predictive value
PPV: positive predictive value
RAAS: renin-angiotensin-aldosterone-system
ROC: receiver operating characteristic
SBP: systolic blood pressure
T2DM: Type 2 diabetes mellitus
TB: tuberculosis
US: United States
VDR: vitamin D receptor
WC: waist circumference
WHO: World Health Organization
