# Supplementary material for: Nutritional assessment among adult patients with suspected or confirmed active tuberculosis disease in rural India
Source: PLoS One. 2020 May 22;15(5):e0233306. doi: 10.1371/journal.pone.0233306 (PMC7244113; doi:10.1371/journal.pone.0233306)
Supplement: S1 Table — (DOCX) [file pone.0233306.s001.docx]

| **S1 Table: Definitions of anemia and related red blood cell indices** | | | | | | | |
| --- | --- | --- | --- | --- | --- | --- | --- |
|  | | **Hematological indicator** | **Age** | | **Male** | **Female** | **Ref** |
| **Anemia** | | Hemoglobin  (g/L) ^b, c^ | ≥ 18 years | Non-pregnant | <130 | <120 | [1] |
|  |  |  |  | Pregnant ^c^ | --- | <110 |  |
| **Severe anemia** | | Hemoglobin  (g/L) ^a, b^ | ≥ 18 years | Non-pregnant | <80 | <80 | [1] |
|  |  |  |  | Pregnant ^c^ | --- | <70 |  |
| **Red blood cell size** | Microcytic | MCV (femtoliters/cell) | --- | | <80 | | [2] |
|  | Normocytic |  | --- | | ≥ 80 and ≤ 100 | |  |
|  | Macrocytic |  | --- | | >100 | |  |
| **Erythrocyte color** (hemoglobin content) | Hypochromic | MCH (picograms/cell) | --- | | <27 | | [2] |
|  | Normochromic |  |  |  | ≥ 27 and ≤ 33 | |  |
|  | Hyperchromic |  |  |  | >33 | |  |
| **Inflammation** | | ESR (mm/hr) | <50 years | | >15 | >20 | [3, 4] |
|  |  |  | ≥50 years and <85 | | >20 | >30 |  |
|  |  |  | ≥85 years | | >30 | >42 |  |
| REF, reference; MCV, mean corpuscular volume; MCH, mean corpuscular hemoglobin; ESR, erythrocyte sedimentation rate  ^a^ Data collection site is <1000 meters above sea level, and therefore there were no hemoglobin adjustments for altitude [1]. Among current smokers, 0.3 g/L was subtracted from hemoglobin.  ^b^ Biologically unlikely hemoglobin values (<25, >200 g/L) considered missing  ^c^ No study participants self-reported as pregnant | | | | | | | |
