# Supplementary material for: Nutritional assessment among adult patients with suspected or confirmed active tuberculosis disease in rural India
Source: PLoS One. 2020 May 22;15(5):e0233306. doi: 10.1371/journal.pone.0233306 (PMC7244113; doi:10.1371/journal.pone.0233306)
Supplement: S2 Table — (DOCX) [file pone.0233306.s002.docx]

| **S2 Table: Additional anthropometric indicators among men and women** ^a^ | | | | |
| --- | --- | --- | --- | --- |
|  | **Total**  **n=834** | **Men**  **n=610 (73.1%)** | **Women**  **n=224 (26.9%)** | **p** ^b^ |
| ***Median (IQR) or n (%)*** | | | | |
| MUAC (cm) | 23.7 (21.5, 26.4) | 23.6 (21.5, 26.2) | 24.0 (21.5, 27.0) | 0.24 ^c^ |
| Low MUAC  (<19.0 cm women,  <20.0 cm men ^e^) | 103 (12.9%) | 86 (14.6%) | 17 (8.1%) | 0.01 ^d^ |
| Triceps skinfold thickness (mm) | 9.3 (5.3, 15.3) | 8.3 (5.0, 13.3) | 12.0 (7.0, 18.5) | <0.01 ^c^ |
| Total body fat (%) | 18.3 (12.7, 25.0) | 15.7 (11.0, 21.0) | 28.0 (22.2, 35.1) | <0.01 ^c^ |
| Trunk fat (%) | 18.4 (11.6, 25.4) | 16.0 (10.3, 22.7) | 26.0 (18.7, 35.1) | <0.01 ^c^ |
| IQR, interquartile range; MUAC, mid-upper arm circumference  ^a^ Among study participants with available anemia (hemoglobin) data (n=834). Covariates with missing observations included: MUAC (n=33), skinfold thickness (n=91), total body fat (n=41), trunk fat (n=41).  ^b^ Comparison between men and women  ^c^ Kruskal Wallis test. Normality assumptions not met based on Shapiro-Wilk test statistic.  ^d^ Likelihood ratio test  ^e^ MUAC cut-off values from previously suggested values [5] | | | | |
