# Supplementary material for: Nutritional assessment among adult patients with suspected or confirmed active tuberculosis disease in rural India
Source: PLoS One. 2020 May 22;15(5):e0233306. doi: 10.1371/journal.pone.0233306 (PMC7244113; doi:10.1371/journal.pone.0233306)
Supplement: S3 Table — (DOCX) [file pone.0233306.s003.docx]

| **S3 Table: Comparison of anthropometric (BMI, WC) screening cut-offs for HbA1c ≥ 5.7%** ^a, b^ | | | | | | | | | |
| --- | --- | --- | --- | --- | --- | --- | --- | --- | --- |
|  | **HbA1c** | |  |  |  |  |  |  |  |
| **BMI** ^c^ (kg/m^2^) | ≥5.7% | <5.7% |  |  | **Sensitivity**  (95% CI) | **Specificity**  (95% CI) | **PPV**  (95% CI) | **NPV**  (95% CI) |  |
| ≥ 25.0 | 10 | 12 | 22 |  |  |  |  |  |  |
| < 25.0 | 64 | 137 | 201 |  | 0.14  (0.06, 0.21) | 0.92  (0.88, 0.96) | 0.45  (0.25, 0.66) | 0.68  (0.62, 0.75) |  |
|  | 74 | 149 | 223 |  |  |  |  |  |  |
|  |  |  |  |  |  |  |  |  |  |
|  |  |  |  |  |  |  |  |  |  |
| ≥ 23.0 | 16 | 22 | 38 |  |  |  |  |  |  |
| < 23.0 | 58 | 127 | 185 |  | 0.22  (0.12, 0.31) | 0.85  (0.80, 0.91) | 0.42  (0.26, 0.58) | 0.69  (0.62, 0.75) |  |
|  | 74 | 149 | 223 |  |  |  |  |  |  |
|  |  |  |  |  |  |  |  |  |  |
| WC ^d^ (cm) |  |  |  |  |  |  |  |  |  |
| ≥ IDF cut-off | 14 | 18 | 32 |  |  |  |  |  |  |
| < IDF cut-off | 61 | 136 | 197 |  | 0.19  (0.10, 0.27) | 0.88  (0.83, 0.93) | 0.44  (0.27, 0.61) | 0.69  (0.63, 0.75) |  |
|  | 75 | 154 | 229 |  |  |  |  |  |  |
|  |  |  |  |  |  |  |  |  |  |
| BMI, body mass index; WC, waist circumference; HbA1c, glycated hemoglobin; PPV, positive predictive value; NPV, negative predictive value; CI, confidence interval; IDF, International Diabetes Federation; WHO, World Health Organization  ^a^ Among study participants with available data (hemoglobin, HbA1c, as well as either BMI [n=223] or WC [n=229]).  ^b^ American Diabetes Association cut-points of HbA1c ≥ 5.7%.  ^c^ WHO classifications (standard and alternative categorization for Asian populations)  ^d^ IDF WC cut-off values among South Asian populations (men ≥80 cm, women ≥90 cm) | | | | | | | | | |
