# Supplementary material for: Nutritional assessment among adult patients with suspected or confirmed active tuberculosis disease in rural India
Source: PLoS One. 2020 May 22;15(5):e0233306. doi: 10.1371/journal.pone.0233306 (PMC7244113; doi:10.1371/journal.pone.0233306)
Supplement: S4 Table — (DOCX) [file pone.0233306.s004.docx]

| **S4 Table: Serum 25-hydroxyvitamin D and waist circumference (n=150)** | | | | | | | | |
| --- | --- | --- | --- | --- | --- | --- | --- | --- |
| **Vitamin D**  **(25[OH]D)** | **WC (continuous [cm]; linear regression)** | | | | **WC (categorical; binomial regression)** ^a, c^ | | | |
|  | Unadjusted | | Adjusted ^b^ | | Unadjusted | | Adjusted ^b^ | |
|  | β (SE) | p | β (SE) | p | RR | 95% CI | aRR | 95% CI |
| Continuous (nmol/L) | -0.08 (0.04) | 0.03 | -0.10 (0.04) | <0.01 | 1.00 ^d^ | 1.00, 1.01 | 1.00 ^d^ | 1.00, 1.01 |
| < 50 nmol/L (Endocrine Society) | 3.15 (1.93) | 0.10 | 3.63 (1.84) | 0.05 | 0.85 | 0.72, 1.01 | 0.82 ^d^ | 0.56, 1.19 |
| Quintiles (low 1 vs 2-5) | 0.24 (2.53) | 0.92 | 0.24 (2.45) | 0.92 | 0.98 | 0.78, 1.22 | 0.95 ^d^ | 0.58, 1.54 |
| 25(OH)D, 25-hydroxyvitamin D; aRR, adjusted risk ratio; RR, risk ratio; WC, waist circumference  **Footnotes**  ^a^ High vs low WC. Cut-offs based on IDF WC cutoffs among South Asian populations.  ^b^ We considered known or suspected risk factors for waist circumference as potential confounders. These potential confounders were included if p<0.25 from univariate regressions (linear or binomial regression model beta coefficients; likelihood ratio tests). Based on a change in estimate approach, covariates were included in the final adjusted model if they changed the estimate by ≥10%. The final covariates for the association of waist circumference (categorical) and vitamin D (quintiles 1 vs 2-5) were utilized in final models in this table; these included: age, sex, active TB disease, anemia, monthly household income.  ^c^ Binomial regression unless otherwise stated  ^d^ Poisson regression due to no model convergence | | | | | | | | |
