# Supplementary material for: Nutritional assessment among adult patients with suspected or confirmed active tuberculosis disease in rural India
Source: PLoS One. 2020 May 22;15(5):e0233306. doi: 10.1371/journal.pone.0233306 (PMC7244113; doi:10.1371/journal.pone.0233306)
Supplement: S5 Table — (DOCX) [file pone.0233306.s005.docx]

| **S5 Table: Serum 25-hydroxyvitamin D and blood pressure (n= 99)** | | | | | | | | | | | |
| --- | --- | --- | --- | --- | --- | --- | --- | --- | --- | --- | --- |
| **Vitamin D**  **(25[OH]D)** | **Systolic blood pressure (continuous; linear regression)** ^b^ | | **Elevated systolic blood pressure (categorical** ≥**140 mmHg; binomial regression)** ^a, b, c^ | | **Diastolic blood pressure (continuous; linear regression)** ^b^ | | | **Elevated diastolic blood pressure (categorical** ≥**90 mmHg; binomial regression)** ^a, b, c^ | | **Abnormal blood pressure (categorical either systolic** ≥**140 mmHg or diastolic** ≥**90 mmHg; Poisson regression)** ^a, b, c^ | |
|  | Adjusted | | | | | | | | | | |
|  | β (SE) | p | RR | 95% CI | β (SE) | p | | RR | 95% CI | RR | 95% CI |
| Continuous (nmol/L) | -0.07 (0.07) | 0.35 | 0.97 ^d^ | 0.94, 1.01 | -0.04 (0.05) | | 0.48 | 1.00 ^d^ | 0.97, 1.03 | 0.97 ^d^ | 0.92, 1.02 |
| < 50 nmol/L (Endocrine Society) | 5.45 (3.28) | 0.10 | 4.00 ^d^ | 0.96, 16.56 | 3.49 (2.41) | | 0.15 | 0.93 ^d^ | 0.27, 3.22 | 4.07 ^d^ | 0.62, 26.60 |
| Quintiles (low 1 vs 2-5) | 0.19 (4.61) | 0.97 | 1.14 ^d^ | 0.18, 7.17 | -0.19 (3.37) | | 0.96 | 0.55 ^d^ | 0.08, 3.66 | 2.55 ^d^ | 0.19, 33.45 |
| 25(OH)D, 25-hydroxyvitamin D  **Footnotes**  ^a^ Elevated blood pressure cut-off values from the National Institutes of Health (NHLBI): https://www.nhlbi.nih.gov/health/health-topics/topics/hbp  ^b^ We considered known or suspected risk factors for blood pressure as potential confounders. These potential confounders were included if p<0.25 from univariate regressions (linear or binomial regression model beta coefficients; likelihood ratio tests). Based on a change in estimate approach, covariates were included in the final adjusted model if they changed the estimate by ≥10%. The final covariates for the associations between hypertension (elevated systolic and/or diastolic blood pressure) and vitamin D (quintiles 1 vs 2-5) were utilized in final models in this table; these included: age, active TB disease, body fat, limb fat, trunk fat, fat free mass, not completing primary education, household income.  ^c^ Binomial regression unless otherwise stated  ^d^ Poisson regression due to no model convergence | | | | | | | | | | | |
